# Clinical course and endocrinological characteristics of prolactinoma in children and adolescents

**DOI:** 10.1186/1687-9856-2013-S1-P197

**Published:** 2013-10-03

**Authors:** Yoo-Mi Kim, Jin-Ho Choi, Beom Hee Lee, Han-Wook Yoo

**Affiliations:** 1 Department of Pediatrics, Asan Medical Center Children’s Hospital, University of Ulsan College of Medicine, Seoul, Korea

## Aim

Prolactinoma is the most prevalent pituitary tumor that accounts for 40% of all pituitary tumors. It is more prevalent in adults with an estimated prevalence of 100/million, however, it is very rare in childhood and adolescents and clinical spectrums and long-term prognosis remain unclear. The most common clinical manifestations have been known to be growth and pubertal disorders. This study investigated clinical and endocrine characteristics, and treatment outcome of prolactinoma in children and adolescents.

## Methods

Six patients (3 males and 3 females) with prolactinoma diagnosed before 18 years of age were included. The diagnosis and relapse of prolactinoma were confirmed by brain magnetic resonance imaging (MRI) and serum prolactin level. The clinical course, endocrinological characteristics, and radiologic findings were reviewed retrospectively.

## Results

The mean age at diagnosis was 12.2 years (range, 7-15 years). Two boys presented with visual disturbance while the other boy complained galactorrhea and stunted growth velocity. All three girls manifested galactorrhea and two of them also displayed secondary amenorrhea. The mean level of prolactin before treatment was 397.5±315.7 ng/mL (106-946). MRI of the pituitary showed macroadenoma in 5 patients, while 1 female had microadenoma. One male and three females were successfully treated with bromocriptine. The mean duration of medical treatment with bromocriptine was 4.8 years (0.1-11). Two girls who had macroadenoma failed to normalize serum prolactin level and therefore underwent transsphenoidal surgery. Galactorhhea disappeared and all girls returned to regular menstrual period. The boy who showed growth retardation came back to normal growth velocity after prolacin level reached normal range. Two boys with visual disturbance underwent an operation at the time of diagnosis being suspicious of craniopharyngioma. After tumor resection they have been under multiple hormone replacement therapy. These two boys had recurrent prolactinoma despite medical treatment and underwent additional surgery and radiation therapy. One boy has not been followed-up and the other has been treated with carbegoline but the fourth operation is under contemplation.

## Conclusion

Children and adolescents with prolactinomas exhibits wide spectrum of clinical presentations. Multimodal treatment such as surgery or radiotherapy may be necessary in some cases who are resistant to dopamine agonists. The patients with high prolactin level and macroadenoma were resistant to medical treatment and had high relapse rate. Long-term follow-up for large cohort of patients with prolactinemia should be needed to delineate clinical course and prognosis.

